# Persistent Symblepharon in an Infant Following Epidemic Keratoconjunctivitis

**Published:** 2016

**Authors:** Sezen AKKAYA, Yelda Buyru OZKURT

**Affiliations:** 1 Fatih Sultan Mehmet Training and Research Hospital, Ophthalmology Department, E-5 uzeri, ust bostanci, Istanbul, Turkey

**Keywords:** Symblepharon, Infant, Epidemic Keratoconjunctivitis

## Abstract

Epidemic keratoconjunctivitis (EKC), caused by certain species D human adenoviruses (Ads), is a highly contagious severe disease involving both the conjunctiva and cornea. The hallmark of this disease is the subepithelial infiltration of leukocytes, which results in corneal opacities that may persist for months or even years.

In this case, of a 6-month-old infant, we report a symblepharon formation, a relatively rare outcome of EKC. In this condition, the palpebral conjunctiva adheres tightly to the bulbar conjunctiva of the eyeball. Our report is the first documentation of a symblepharon formation in an infant. Only two similar cases have been reported to date; therefore, a detailed description is of considerable interest to ophthalmologists. This is particularly interesting since a previous publication has associated symblepharon formation with an adenovirus infection, which is not usually involved in EKC. The development of a symblepharon following EKC is rare in infants. Since topical treatment cannot be applied due to severe eyelid edema, oral steroid therapy can be administered with pediatric consultation and meticulous monitoring.

## INTRODUCTION

Epidemic keratoconjunctivitis (EKC) is considered a distressing contagious infection of the ocular surface (1). Adenoviral conjunctivitis is epidemic in adult and pediatric populations (2). The transmission of adenovirus occurs primarily through hand-to-eye contact, respiratory droplets, eye secretions, and contact with healthcare providers and their medical equipment. The most frequent manifestation of an ocular adenoviral infection is epidemic keratoconjunctivitis, followed by pharyngoconjunctival fever. Epidemic keratoconjunctivitis is the most rigorous form of disease caused by adenovirus and presents with watery discharge, chemosis, hyperemia, and unilateral lymphadenopathy (2).

Moreover, the incubation period fluctuates from 4 hours to 3 weeks and the symptoms may last for 7-21 days. As a biphasic disease, adenoviral conjunctivitis starts with an infective period followed by an inflammatory phase. The inflammatory period commences around a week followed by the initial infection as the virus continues to shed (3). It is usually diagnosed on the basis of symptoms and clinical manifestations. Although Laboratory evaluation is mostly not necessary, it can be beneficial for the diagnosis (4).

Keratoconjunctivitis may be severe, sometimes causing substantial morbidity. The distinguishing characteristic of EKC is involvement of the whole ocular surface area, along with the surface of the conjunctival and corneal epithelium. In severe cases, pseudomembranes and symblepharon may form and multifocal subepithelial infiltrates may reduce visual acuity for a long duration (1). The configuration of pseudomembranes includes sheets of fibrin exudates lacking blood vessels. It is a frequent complication in EKC, particularly in infections associated with specific serotypes, such as 8, 19, and 37 (2). This suggests that true conjunctival membranes can and do form in EKC. When removed, these true membranes can cause bleeding, while their persistence may cause subepithelial fibrosis and the formation of a symblepharon.

Several topical treatment methods have been used for EKC. The majority of them are prophylactic, including preservative-free antibiotics and artificial tears. Some authors have found that 2.5% povidone iodine is an effective treatment for EKC (5). Viral protein remnants frequently persist on the corneal surface of Bowman’s layer for a long period and may form subepithelial infiltrates, which are challenging to treat. Cyclosporin A eye drops are a respectable option for the low risk cases. The use of topical steroids has potential disadvantageous; however, using them may be considered at any stage of the disease (3).

## CASE REPORT

**Figure 1:**
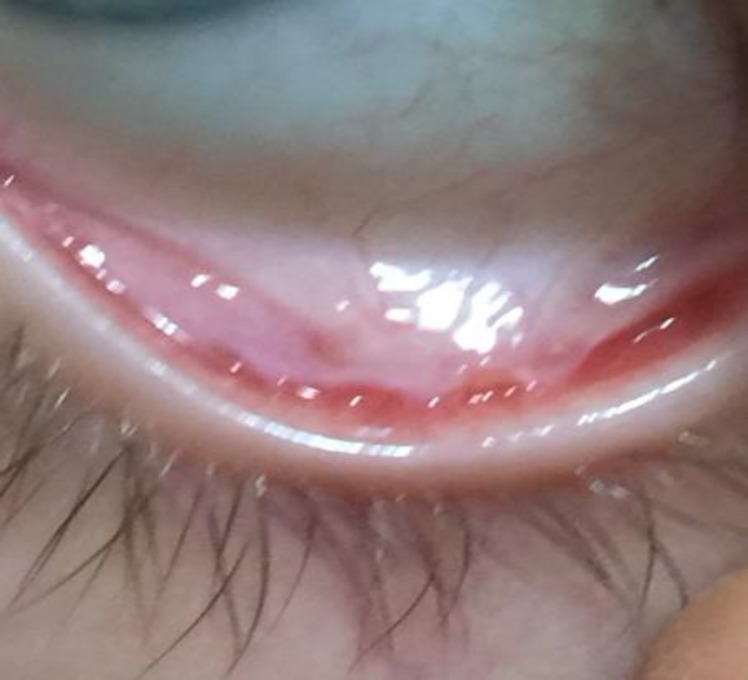
Persistent symblepharon after 3 years following treatment.

We obtained signed statement from the patient's parents and our hospital ethical committee, which authorized the use of the patient's personal and/or medical information for the publication of our study. Multiple cases of EKC had been reported in our hospital. The polymerase chain reaction findings for one patient showed that the EKC was caused by adenovirus serotype 19. A 6-month-old infant presented with EKC in both eyes. His 1- and 3-month postpartum eye exams had normal findings. The patient’s mother had EKC 20 days prior and was treated with topical drops at our clinic. The infection in the son began one day prior to the hospital visit. The patient underwent inspection and direct ophthalmoscopy, which showed bilateral epiphora, chemosis, severe eyelid edema, and preauricular lymphadenopathy. The eyelid was forced open and there was no corneal staining with fluorescein. Topical preservative-free antibiotics, artificial tears, and loteprednol etabonate drops were prescribed. In addition, conjunctival irrigation was performed with 2.5% povidone iodine. The patient failed to return for a 3-day follow-up visit, but returned 21 days after the examination, when the mother said that the treatment could not be administered because of the edema and pain. Examination showed improvement of the eyelid edema and normal corneas; however, there were bilateral conjunctival scars and a symblepharon formation in the lower left eyelid between the temporal palpebral conjunctiva and bulbar conjunctiva. Eye movements were not restricted. The patient was treated with weak steroids and artificial tear drops, but the symblepharon persisted for at least three years (Figure 1).

## DISCUSSION

The main concern in the treatment of cases with definite or suspected EKC is meticulous hygiene practices mainly because there is still no efficient medication for this disease. No virostatic agent has been shown to affect the course of the virus (4). In mild cases, a prescription for artificial tears, cold compresses, and vasoconstrictors may be effective. In more severe cases, topical steroids should be prescribed when the disease causes the formation of a pseudomembrane. Based on the severity of the case, an ophthalmologist may remove pseudomembranes on an individual basis (3).

Hammer et al. (6) presented two case reports in which patients with EKC developed symblepharon during the course of infection. That was the first report of symblepharon formations associated with adenovirus type 2. The first case involved an 82-year-old woman who had previously undergone a keratoplasty operation. In that case, the symblepharon formation was completely reversed after continued lysis with a glass rod. The second case involved a healthy 47-year-old woman with no previous ocular disease. The symblepharon formation remained unchanged during the 18 months of observation. Initial treatment for both patients was topical antibiotics and steroids (6). Our report is the first of a symblepharon formation in an infant. We treated the patient with topical preservative-free antibiotics, artificial tears, loteprednol etabonate for severe eyelid edema, and irrigation of the conjunctiva with 2.5% povidone iodine. 

Topical steroids are crucial when a pseudomembrane forms. Eye drops with 0.12% prednisolone acetate, 0.1% fluorometholone, 1% rimexolone, dexamethasone, and 0.001% loteprednol etabonate may be applied, and eye drops with 1.0% prednisolone or 0.5% loteprednol etabonate are recommended while keratitis is present (7). Nonsteroidal anti-inflammatory drugs (NSAIDs) or oral steroids may be prescribed for symptomatic relief in younger patients less than 3 years of age with severe EKC and inflammatory ptosis (7). It should be noted that in our case, we treated conservatively and did not use an oral steroid or any nonsteroidal medications.

Studies showed that virostatic agents such as trifluridine, vidarabine, and ganciclovir may be effective against adenovirus; however, the latest studies regarding their efficacy for the treatment of adenoviral conjunctivitis are still notorious (2). Povidone-iodine as a broad-spectrum antiseptic may be effective against free adenovirus, but research has shown that it is less efficient against intracellular adenovirus (5).

Irrigation of conjunctiva with 2.5% povidone iodine is an effective treatment for adenoviral conjunctivitis in infants (5). Tunay et al. report significantly lower clinical scores and reduced recovery time in infants irrigated with a 2.5% povidone iodine solution when compared to those without conjunctival irrigation. We recommend calling the parents of infant patients every three days to receive an update on symptoms; this can enable repeat irrigation if required. Unfortunately, our patient did not return for a 3-day check-up; thus, we were unable to repeat irrigation.

Short-term oral steroid therapy rapidly improves eyelid edema, inflammatory ptosis, and corneal erosion. Kim et al. (7) recommend prescribing oral steroids, especially when there is poor application of eye drops in patients with EKC who are younger than 3 years of age. Based on the severity of the symptoms, they initially administered a 0.6 mg/kg dose of oral prednisolone, which was increased up to 1.0 mg/kg at the pediatrician’s discretion. Short-term oral steroids were discontinued without tapering as soon as the symptoms improved. Pseudomembranes were removed as soon as possible; however, removal was difficult in non-compliant patients due to eyelid swelling and ocular pain. Pseudomembrane removal was promising 2 to 3 days following initiation of oral steroid therapy. However, oral steroids have some side effects such as the reduction of the growth rate by decreasing the function of the hypothalamus–pituitary–adrenal axis, reduction in bone density, and the development of Cushing syndrome, hypertension, or diabetes (7).

We did not administer oral steroids in our case due to the danger of the side effects in a 6-month-old infant. However, based on the symblepharon formation, we believe that oral steroids would have been a suitable treatment given that there would be a pediatric consultation and very strict follow-up. Steroids would have been particularly useful since we were unable to effectively administer topical treatment due to severe eyelid edema. However, short-term follow-up examinations and pediatric consultations are required when administering oral steroids.

## References

[1] Gunay M, Celik G, Con R (2015). Treatment for retinopathy of prematurity in an infant with adenoviral conjunctivitis. Case Rep Pediatr.

[2] Jhanji V, Chan TC, Li EY, Agarwal K, Vajpayee RB (2015 ). Adenoviral keratoconjunctivitis. Surv Ophthalmol.

[3] Kaufman HE (2011). Adenovirus advances: new diagnostic and therapeutic options. Curr Opin Ophthalmol.

[4] Udeh BL, Schneider JE, Ohsfeldt RL (2008). Cost effectiveness of a point-of-care test for adenoviral conjunctivitis. Am J Med Sci.

[5] Özen Tunay Z, Ozdemir O, Petricli IS (2015). Povidone iodine in the treatment of adenoviral conjunctivitis in infants. Cutan Ocul Toxicol.

[6] Hammer LH, Perry HD, Donnenfeld ED, Rahn EK (1990). Symblepharon formation in epidemic keratoconjunctivitis. Cornea.

[7] Kim SY, Chung YK, Lee YC, Kim SY (2015). Oral steroid therapy as an adjuvant treatment for severe epidemic keratoconjunctivitis in patients younger than 3 years. Cornea.

